# Loss-of-function alleles of *ZmPLD3* cause haploid induction in maize

**DOI:** 10.1038/s41477-021-01037-2

**Published:** 2021-12-09

**Authors:** Yuan Li, Zhen Lin, Yang Yue, Haiming Zhao, Xiaohong Fei, Lizhu E., Chenxu Liu, Shaojiang Chen, Jinsheng Lai, Weibin Song

**Affiliations:** 1 State Key Laboratory of Plant Physiology and Biochemistry, China Agricultural University, Beijing, P. R. China; 2 National Maize Improvement Center, Department of Plant Genetics and Breeding, China Agricultural University, Beijing, P. R. China; 3 Center for Crop Functional Genomics and Molecular Breeding, China Agricultural University, Beijing, P. R. China; 4 Sanya Institute of China Agricultural University, Sanya, P. R. China; 5 Hainan Yazhou Bay Seed Laboratory, Sanya, P. R. China; 6 Longping Agriculture Science Co. Ltd., Beijing, P. R. China

**Keywords:** Agricultural genetics, Plant breeding

## Abstract

Doubled haploid technology has been widely applied to multiple plant species and is recognized as one of the most important technologies for improving crop breeding efficiency. Although mutations in *MATRILINEAL/Zea mays PHOSPHOLIPASE A1/NOT LIKE DAD* (*MTL/ZmPLA1/NLD*) and *Zea mays DOMAIN OF UNKNOWN FUNCTION 679 MEMBRANE PROTEIN* (*ZmDMP*) have been shown to generate haploids in maize, knowledge of the genetic basis of haploid induction (HI) remains incomplete. Therefore, cloning of new genes underlying HI is important for further elucidating its genetic architecture. Here, we found that loss-of-function mutations of *Zea mays PHOSPHOLIPASE D3* (*ZmPLD3*), one of the members from the phospholipase D subfamily, could trigger maternal HI in maize. *ZmPLD3* was identified through a reverse genetic strategy based on analysis of pollen-specifically expressed phospholipases, followed by validation through the clustered regularly interspaced short palindromic repeats/CRISPR-associated protein 9 (CRISPR–Cas9) system. Mutations of *ZmPLD3* resulted in a haploid induction rate (HIR) similar to that of *mtl/zmpla1/nld* and showed synergistic effects rather than functional redundancy on tripling the HIR (from 1.19% to 4.13%) in the presence of *mtl/zmpla1/nld*. RNA-seq profiling of mature pollen indicated that a large number of pollen-specific differentially expressed genes were enriched in processes related to gametogenesis development, such as pollen tube development and cell communication, during the double-fertilization process. In addition, *ZmPLD3* is highly conserved among cereals, highlighting the potential application of these in vivo haploid-inducer lines for other important crop plant species. Collectively, our discovery identifies a novel gene underlying in vivo maternal HI and provides possibility of breeding haploid inducers with further improved HIR.

## Abstract (web summary)

This study revealed a novel regulator, ZmPLD3, for maternal haploid induction in maize. Loss-of-function mutations of *ZmPLD3* showed an induction rate similar to that of *mtl/zmpla1/nld* and induced haploids in a synergistic manner with the *mtl* mutations.

## Main

Doubled haploid (DH) technology based on in vivo haploid induction (HI) increases the breeding efficiency by enabling the rapid production of homozygous inbred lines, which has been widely applied in modern maize breeding^1^. Via the selective breeding of the ancestral haploid-inducer Stock 6 (ref. ^2^), modern plant breeders have created a variety of haploid inducers that have high haploid induction rates (HIR) and excellent agronomic traits, including UH400 (ref. ^3^), RWS (ref. ^4^) and CAU5 (ref. ^5^). These HI lines provide an effective method for the mass production of DH lines in commercial maize breeding programmes. Because of its vital application value in modern breeding, the genetic factors controlling this phenomenon have been widely investigated^3,6^.

To date, many linkage analyses and genome-wide association studies have been performed on the identification of quantitative trait loci (QTLs) to unveil the genetic architecture of HI. Several QTLs related to HIR were identified by using four biparental populations, among which *quantitative haploid induction rate 1* (*qhir1*) in bin 1.04 and *quantitative haploid induction rate 8* (*qhir8*) in bin 9.01 explained ~66% and ~20% of the genetic variance for HI, respectively^3^. Fine mapping was conducted to narrow the two major QTLs to smaller genomic intervals of 243 kilobases (kb) in bin 1.04 (ref. ^7^) and 789 kb in bin 9.01 (ref. ^8^). In addition to the two major QTLs responsible for HI, other QTLs with weaker effects have also been identified^3^, which suggested that the genetic architecture of HI is much more complex.

In 2017, three different research groups found that the causative allele for HI of *qhir1* in the Stock 6 background was a 4-base pairs (bp) insertion in the fourth exon of *MTL/ZmPLA1/NLD*^9–11^. Given the conservation of *MTL/ZmPLA1/NLD* in cereals, knockout of *MTL/ZmPLA1/NLD* orthologues in rice^12^ and wheat^13^ also triggered HI. It has been shown that *MTL/ZmPLA1/NLD*, encoded a pollen-specific patatin-like phospholipase A expressing specifically in vegetative cell but not sperm cell^14^. Further analysis of subcellular localization revealed that MTL/ZmPLA1/NLD targeted the endo-plasma membrane, a specific membrane derived from vegetative cell that surrounds the two sperm cells in pollen^14–16^. These results implied that phospholipases highly expressed in pollen might play an important role in sexual reproduction. According to the different sites of bond cleavage in their respective phospholipid substrates^17^, plant phospholipases have been classified as phospholipase A (PLA), phospholipase C (PLC) and phospholipase D (PLD). Previous studies have shown that multiple phospholipase-mediated membrane lipid metabolism processes are involved in the modulation of pollen development^18–24^. *Arabidopsis* DEFECTIVE IN ANTHER DEHISCENCE1 (DAD1), a chloroplastic PLA, was found to be required for pollen maturation, anther dehiscence and flowering, and associated with the accumulation of jasmonic acid in flower buds^18^. Furthermore, mutants of *NON-SPECIFIC PHOSPHOLIPASE C2* (*NPC2*) and *NON-SPECIFIC PHOSPHOLIPASE C2* (*NPC6*) in *Arabidopsis thaliana* presented defective pollen tube growth caused by the suppression of phospholipid hydrolysis and triacylglycerol biosynthesis^24^. PLD‐produced phosphatidic acid plays a key role in polar expansion of pollen tubes, suggesting that PLD-dependent signalling is vital during tip growth and plant cell expansion^19^.

Recently, Zhong et al.^25^ identified that a single-nucleotide change in *ZmDMP*, which encodes a DUF679 domain-containing protein, was present in the causative allele for HI of *qhir8*. Mutations in *ZmDMP* resulted in HI with an HIR of 0.1–0.3%, and the HIR increased to 6–10% in the presence of *mtl/zmpla1/nld*, which suggested that more than one pathway might be involved in the high HIR observed in commercial haploid-inducer lines. Furthermore, mutations in *Arabidopsis* orthologous genes *AtDMP8* and *AtDMP9* could also trigger maternal haploids^26^, whereas no functional *MTL/ZmPLA1/NLD* orthologous genes were identified in dicots. These findings implied that HI was triggered by genes in different pathways and pyramiding these causative factors together could improve the HIR sharply. Isolating new genes required for HI will contribute to breeding haploid-inducer lines with high HIR, as well as elucidating the mechanisms underlying HI.

In our present study, we demonstrated that knockout of ZmPLD3, a PLD expressed specifically in pollen, triggered HI in maize. Moreover, it enhanced HIR by threefold in the presence of *mtl/zmpla1/nld*. In addition, pollen transcriptome analysis of *zmpld3* and *mtl/zmpla1/nld* indicated that plenty of pollen-specific genes related to cell communication were differentially expressed in these mutants. Collectively, these findings suggested that ZmPLD3 acted as a synergistic factor together with MTL/ZmPLA1/NLD in HI.

## Results

### *ZmPLD3* encodes a phospholipase expressed specifically in pollen

**Fig. 1:**
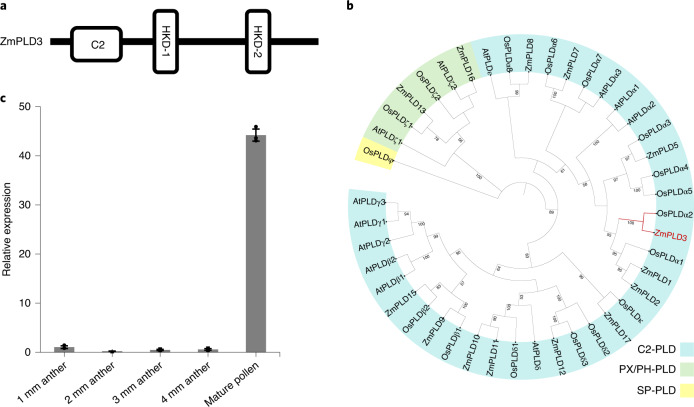
Expression characteristics of ZmPLD3. **a**, Schematic diagram of conserved domains in the ZmPLD3 protein, predicted by Pfam and SMART. The C2 box refers to protein kinase C-conserved region 2; the HKD (HxKxxxxD) boxes refer to conserved catalytic regions. **b**, Phylogenic analysis of PLD in maize, rice and *A. thaliana*. The C2-PLD, PXPH-PLD and SP-PLD subfamilies are indicated by blue, green and yellow backgrounds, respectively. **c**, Relative expression analysis of *ZmPLD3* of anther and mature pollen at different developmental stages from the wild type was determined by qRT–PCR. The values are the means ± s.d. of three biologically independent samples (each sample involved three technical repetitions).

**Extended Data Fig. 1:**
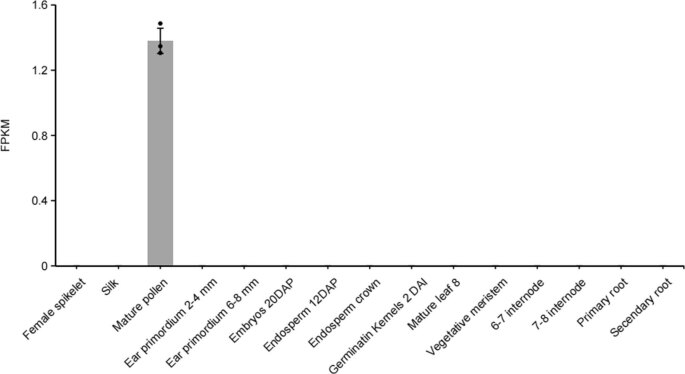
Expression profiles of *ZmPLD3*. Transcript levels (fragments per kilobase of exon model per million mapped fragments (FPKM)) of *ZmPLD3* among different tissues based on previously published RNA sequencing (RNA-seq) data. The values are the means ± s.d. of three biologically independent samples (except for the vegetative meristem samples).

**Extended Data Fig. 2:**
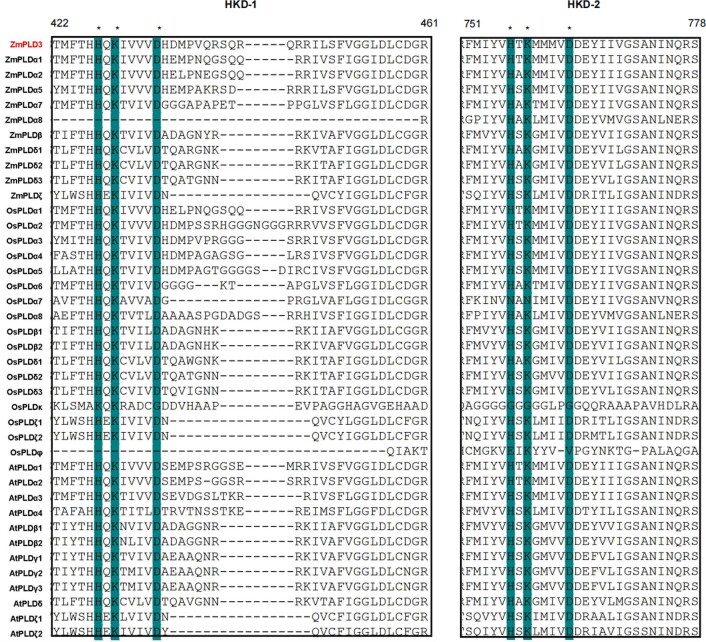
HKD motifs of phospholipase D in maize, rice and *Arabidopsis thaliana*. Multiple alignment of HKD motifs of PLDs in maize, rice and *Arabidopsis thaliana*. The shading and asterisks denote catalytic triads.

**Extended Data Fig. 3:**
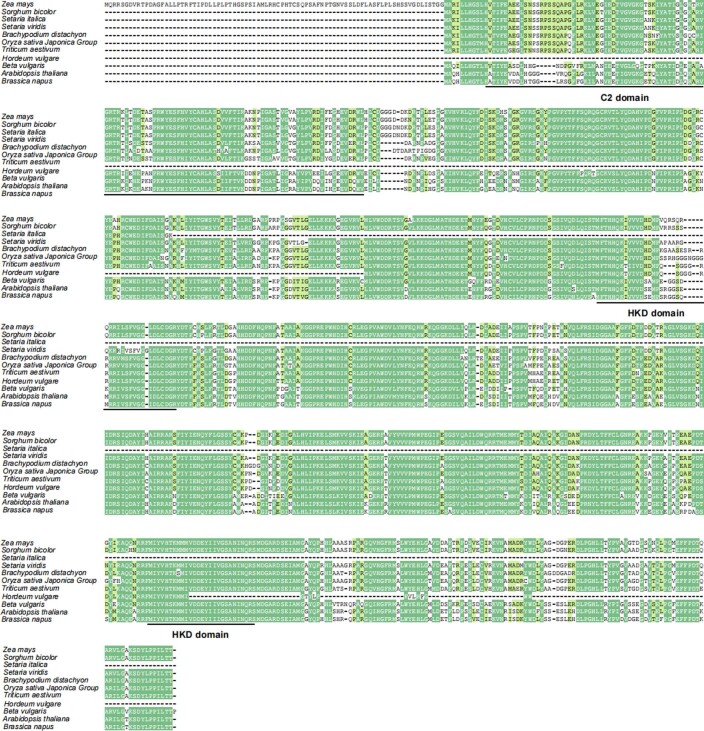
Multiple alignment of ZmPLD3 orthologs in eight monocots and three dicots. The amino acid sequences of ZmPLD3 and its orthologs were downloaded from the website http://www.gramene.org/. Multiple alignments including *Zea mays* (Zm00001d037643), *Sorghum bicolor* (SORBI_3009G062600, 96% sequence identity to that of ZmPLD3), *Setaria viridis* (SEVIR_3G102400v2, 92% identity), *Setaria italica* (SETIT_024724mg, 90% identity), *Oryza sativa Japonica Group* (Os05g0171000, 86% identity), *Triticum aestivum* (TraesCS1A02G115300, 84% identity), *Brachypodium distachyon* (BRADI_2g34290v3, 83% identity), *Hordeum vulgare* (HORVU1Hr1G025370, 82% identity), *Beta vulgaris* (BVRB_9g219660, 75% identity), *Brassica napus* (BnaC05g37540D, 71% identity), and *Arabidopsis thaliana* (AT3G15730, 71% identity) were performed. The dark- and light-green backgrounds indicate increasingly conserved positions. Three conserved domains are indicated by underlines.

To characterize the effects of phospholipase-mediated HI in maize, we used RNA-seq data^27^ from different tissues of maize B73 to identify pollen-specific members of this gene family expressed specifically in pollen (Supplementary Tables 1 and 2). We found that only one member (*ZmPLD3*) was expressed specifically in pollen (Extended Data Fig. 1) and significantly upregulated in *mtl/zmpla1/nld*^9^, which suggested that *ZmPLD3* played a role similar to that of *MTL/ZmPLA1/NLD*. Quantitative PCR with reverse transcription (qRT–PCR) analysis revealed that *ZmPLD3* was highly expressed in mature pollen compared with anthers at different developmental stages (Fig. 1c), suggesting that *ZmPLD3* might play a role late in pollen developmental stage. *ZmPLD3* encodes a putative PLD, which is named for its hydrolytic active-site region (HKD motif, HxKxxxxD)^17^. Further analysis indicated that two HKD domains are present in ZmPLD3 (Fig. 1a and Extended Data Fig. 2). In addition, we aligned the full-length sequences of PLD family proteins and constructed a phylogenetic tree for all members from the genomes of *Zea mays*, *Oryza sativa* and *A. thaliana*, respectively (Fig. 1b, Extended Data Fig. 2 and Supplementary Table 3). On the basis of their conserved domains and phylogenetic relationships, all these PLDs were classified into three clades (C2-PLD, PXPH-PLD and SP-PLD). ZmPLD3 was grouped into the C2-PLD subfamily, which was consistent with its predicted C2 domain that binds to Ca^2+^ cofactors^17^. On the basis of their molecular and enzymatic characteristics, PLD proteins could also be divided into different clades^28^ and phylogenetic analysis showed that ZmPLD3 clustered on a clade together with the α subfamily of PLD in *O. sativa* and *A. thaliana*, of which orthologous genes in *Brassica napus* were reported to be involved in reproductive development^22^. The amino acid alignment of ZmPLD3 orthologues in several species showed that ZmPLD3 was highly conserved among cereal crop species (Extended Data Fig. 3). The integration of expression data and the phylogenetic data suggested that pollen-specific *ZmPLD3* might have a unique function in male reproductive processes. Thus, we considered *ZmPLD3* as a candidate gene responsible for HI for following research.

### Knockout of *ZmPLD3* triggered maternal HI in maize

**Fig. 2:**
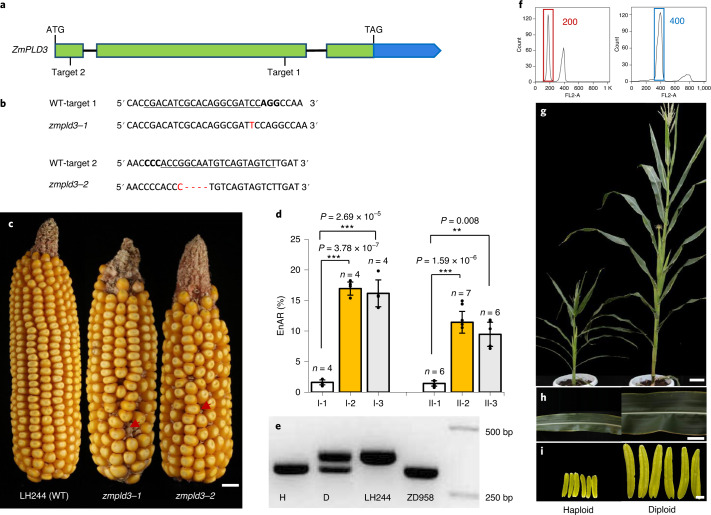
Haploid induction phenotypes of *ZmPLD3* mutants. **a**, *ZmPLD3* structure with the CRISPR-Cas9 target sites shown. **b**, The insertion and deletion sites of two allelic mutations (*zmpld3-1* and *zmpld3-2*) are shown in the alignment comparison with the wild-type (WT) sequence. **c**, Phenotype of the transgenic receptor LH244 (WT) and two allelic mutations (*zmpld3-1* and *zmpld3-2*) of ears produced via self-crossing. The arrows indicate aborted kernels. Scale bars, 1 cm. **d**, The rates of endosperm aborted kernels (EnAR) were significantly different between the knockout lines and WT in both the self-pollinated and crossed ears. I-1, transgenic receptor line (WT); I-2, *zmpld3-1*; I-3, *zmpld3-2*; II-1, ZD958 × transgenic receptor line (WT); II-2, ZD958 × *zmpld3-1*; II-3, ZD958 × *zmpld3-2*. Value *n* indicates the number of ears used for evaluating the HIR and the EnAR and the seed setting rate of each genotype. The plot values are the means ± s.d.; **P < 0.01, ***P < 0.001 (two-sided Mann–Whitney test). **e**, PCR products of haploid and diploid plants with polymorphic markers between the transgenic receptor line and tester. A DNA marker is shown in the far right lane. H, haploid; D, diploid; LH244, transgenic receptor; ZD958, hybrid tester. **f–i**, Flow cytometry results (**f**), overall phenotypes (**g**), 12th leaves (**h**) and anthers (**i**) of representative haploid (left) and diploid (right) plants among the progeny of ZD958 pollinated by *ZmPLD3* knockout plants (as males). Scale bars, 10 cm (**f**), 2 cm (**g**) and 1 mm (**h**). In **e**–**i**, experiments were repeated 206 times and similar results were obtained﻿. Source data

**Extended Data Fig. 4:**
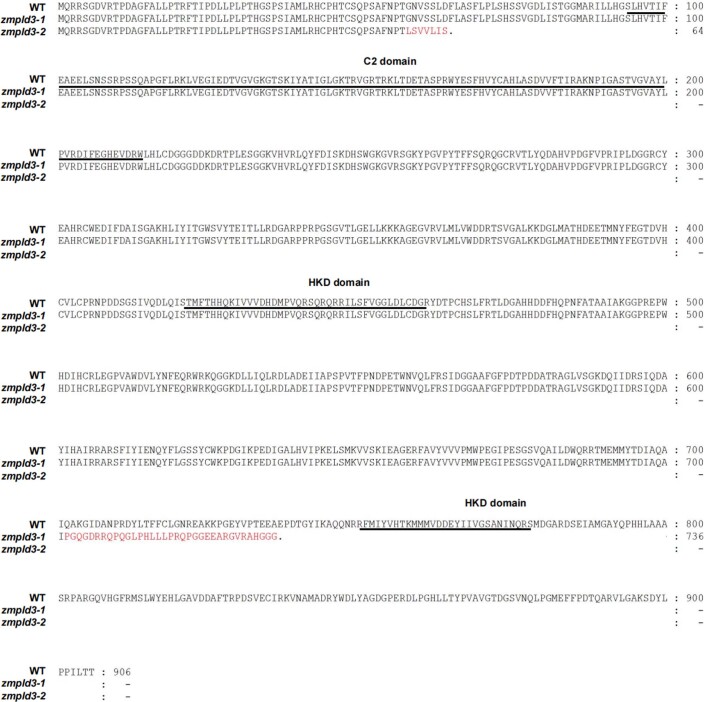
Predicted protein sequence of ZmPLD3 in the wild type and mutants (*zmpld3-1*, *zmpld3-2*). An amino acid alignment of ZmPLD3 predicted the protein sequence in LH244 (WT), with the predicted sequence of the *zmpld3* allele found in the mutants of *zmpld3-1* and *zmpld3-2*. Altered amino acids are shown in red, three conserved domains are indicated by underlines, and stop codons are indicated with full stops.

**Extended Data Fig. 5:**
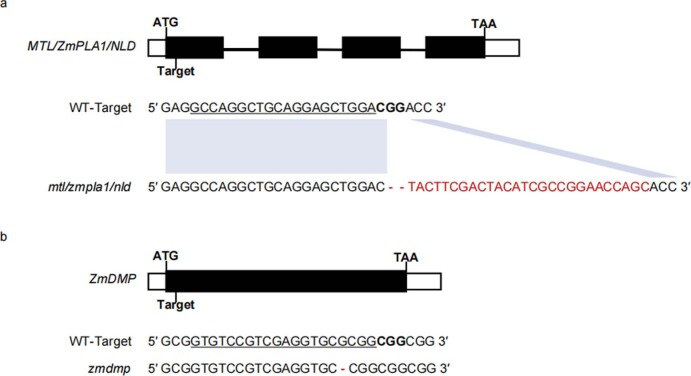
CRISPR-Cas9-mediated target mutagenesis of *MTL/ZmPLA1/NLD* and *ZmDMP*. a, *MTL/ZmPLA1/NLD* structure with the CRISPR-Cas9 target sites shown. *mtl/zmpla1/nld* had both a 2-bp deletion and a 27-bp insertion in its target region, causing 89 changed amino acids starting from the mutation site and resulting in premature translation termination. b, *ZmDMP* structure with the CRISPR-Cas9 target sites shown. *zmdmp* had a 1-bp deletion in its target region, causing 41 changed amino acids starting from the mutation site and resulting in premature translation termination. Mutation sites of the knockout lines are shown in the alignment comparison with the wild-type (WT) sequence, respectively. The target sequences are underlined, with the protospacer-adjacent motif shown in bold type. Insertions are shown in dark red and deletions are shown by dark red dashes.

To further investigate its function, we used CRISPR-Cas9 system to knockout *ZmPLD3*. For CRISPR-Cas9 vector construction, we designed two target sites in different exons of *ZmPLD3*, one (Target 1) within the second exon, which contains predicted conserved domains, and the other (Target 2) within the first exon (Fig. 2a). The vector construct was subsequently transformed into the inbred line LH244 (a non-inducer) to generate mutant lines. Two mutant lines, *zmpld3-1* and *zmpld3-2*, were screened for further study*.* Gene *zmpld3-1* had a 1-bp insertion in its target region, changing 35 altered amino acids behind the insertion site and truncating 170 amino acids in the protein, whereas *zmpld3-2* had both a 5-bp deletion and a 1-bp insertion in its target region, causing seven changed amino acids starting from the mutation site and resulting in premature translation termination (Fig. 2b and Extended Data Fig. 4). Meanwhile, we exploited CRISPR-Cas9 system to generate single-gene mutants of *mtl/zmpla1/nld* and *zmdmp* to evaluate their HI efficiency in LH244 genetic background (Extended Data Fig. 5). It was worth noting that *zmpld3* mutation showed severe segregation distortion in the population derived from the selfed progeny of heterozygous single mutant, which was similar to that of *mtl/zmpla1/nld* mutation (Supplementary Table 4).

**Fig. 3:**
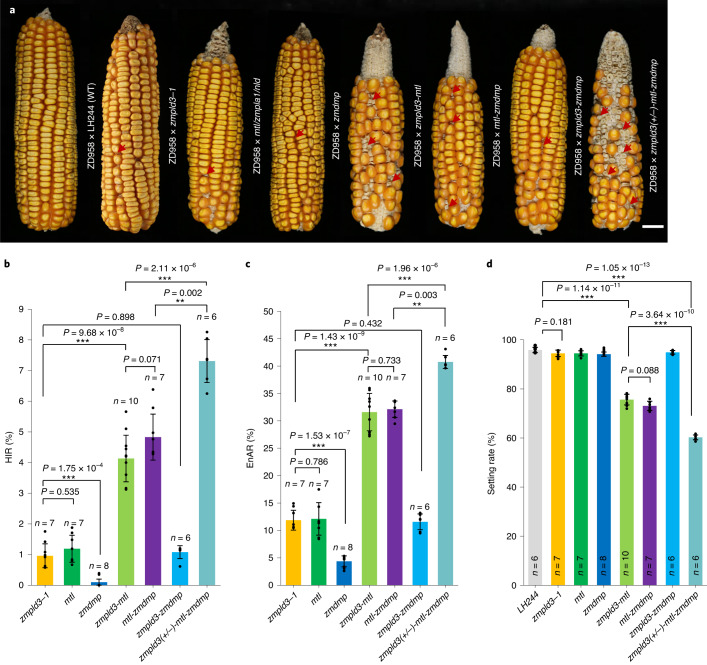
The synergistic effects of *zmpld3* and *mtl/zmpla1/nld* on haploid induction phenotypes. **a**, Phenotypes of ZD958 ears pollinated by LH244 (wild type), *zmpld3-1*, *mtl/zmpla1/nld*, *zmdmp*, *zmpld3-mtl*, *mtl-zmdmp*, *zmpld3-zmdmp* or *zmpld3*(+/–)*-mtl-zmdmp*. The arrows indicate aborted kernels. Scale bars, 2 cm. **b**, HIR of ZD958 ears pollinated by *zmpld3-1*, *mtl/zmpla1/nld*, *zmdmp*, *zmpld3-mtl*, *mtl-zmdmp*, *zmpld3-zmdmp* or *zmpld3*(+/–)*-mtl-zmdmp*. Value *n* indicates the number of ears used for evaluating the HIR and the EnAR and the seed setting rate of each genotype. **c**, The EnAR of ZD958 ears pollinated by *zmpld3-1*, *mtl/zmpla1/nld*, *zmdmp*, *zmpld3-mtl*, *mtl-zmdmp*, *zmpld3-zmdmp* or *zmpld3*(+/–)*-mtl-zmdmp*. **d**, The seed setting rate of ZD958 ears pollinated by LH244, *zmpld3-1*, *mtl/zmpla1/nld*, *zmdmp*, *zmpld3-mtl*, *mtl-zmdmp*, *zmpld3-zmdmp* or *zmpld3*(+/–)*-mtl-zmdmp*. The values are the means ± s.d.; *P < 0.05, **P < 0.01, ***P < 0.001 (two-sided Mann–Whitney test) (**b**–**d**).

**Extended Data Fig. 6:**
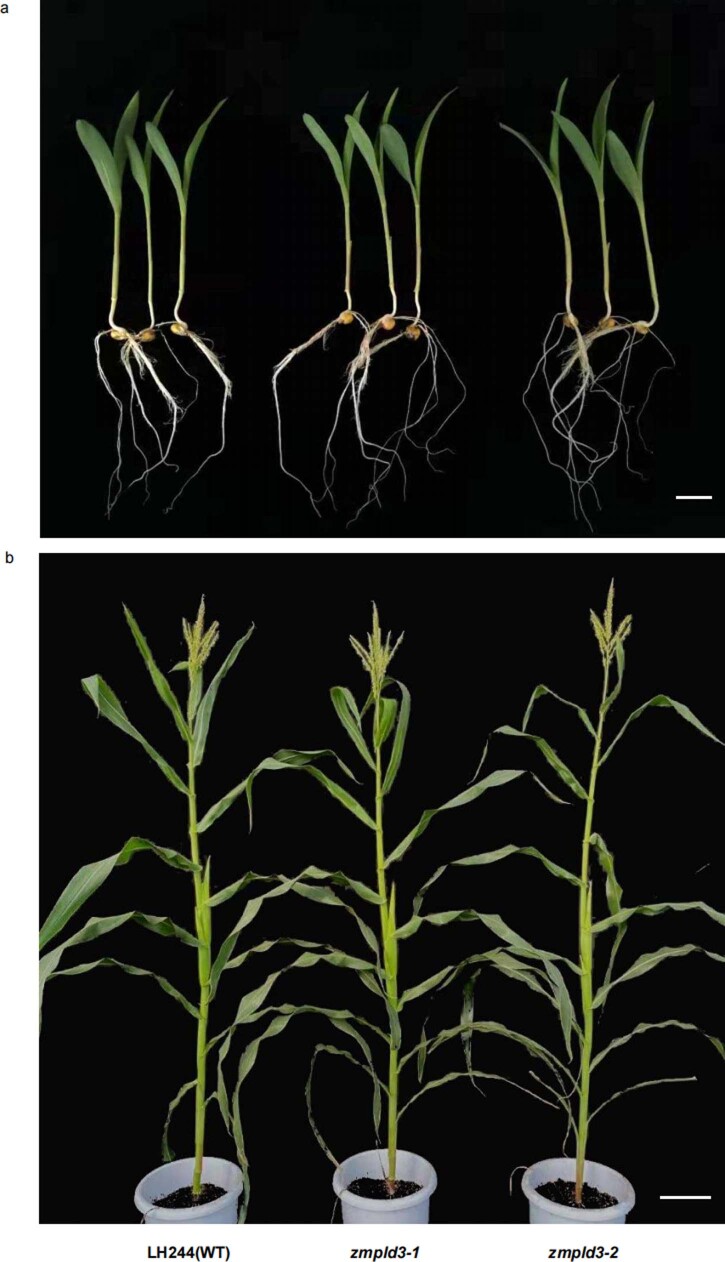
The morphological phenotypes of seedlings and mature plants did not show obvious differences between the wild type and mutants (*zmpld3-1*, *zmpld3-2*). Seedlings at seven days after sowing (a) and mature plants producing pollen (b) of LH244 and the mutants of *zmpld3-1* and *zmpld3-2* are shown. Scale bars, 1 cm (a) and 15 cm (b).

**Extended Data Fig. 7:**
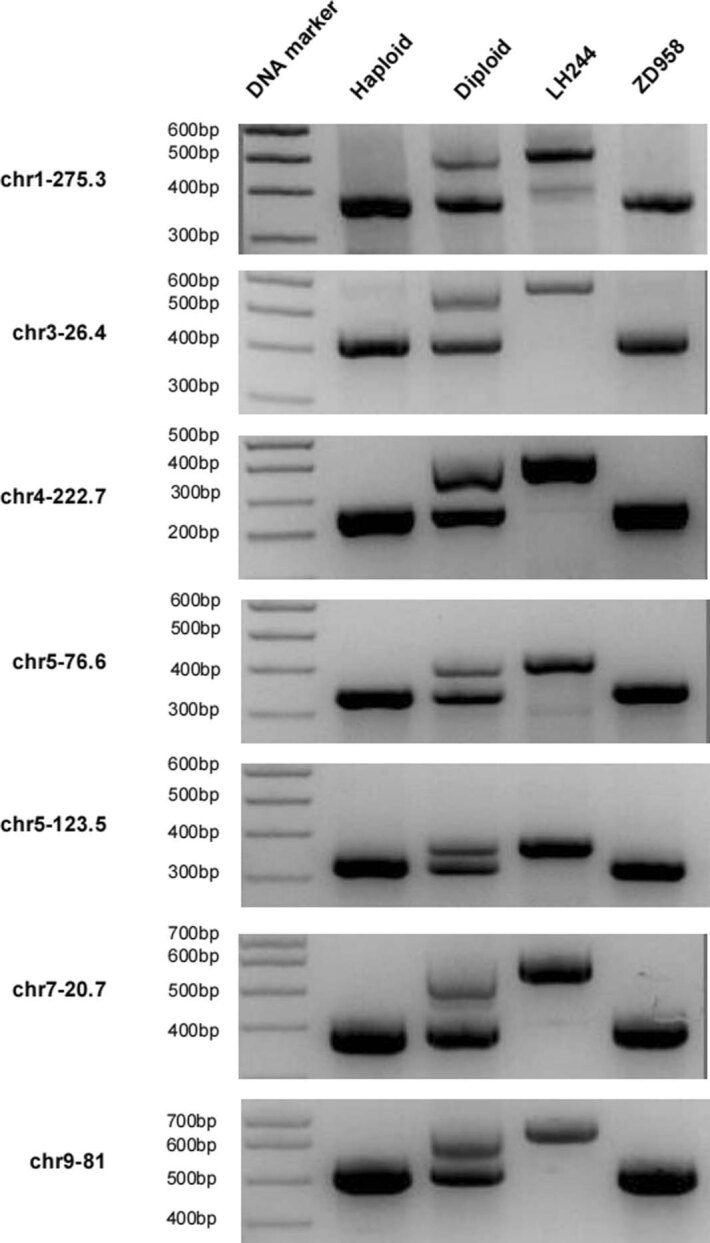
Identification of haploids via polymorphic molecular markers. The lanes from left to right show DNA markers and band performance of haploid and diploid progeny as well as two parents, with 7 molecular markers. Experiments were repeated 206 times and similar results were obtained. Source data

**Extended Data Fig. 8:**
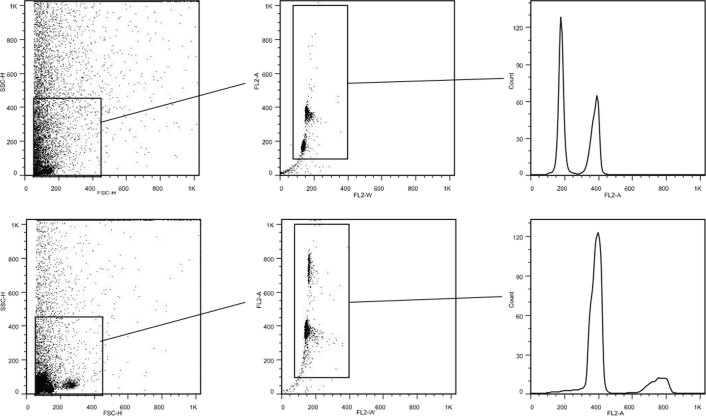
Gating strategies of flow cytometry used for ploidy identification. All the haploid candidates were screened by the same gating strategies of flow cytometry. Experiments were repeated 231 times and similar results were obtained (the flow cytometry results of haploids were as the top panel, whereas the flow cytometry results of diploids were as the bottom panel).

Both the mutants and wild-type (WT) LH244 were grown in a greenhouse and no obvious differences were observed in their morphological phenotypes from the seedling stage to the mature stage during pollen scattering (Extended Data Fig. 6). However, their self-pollinated ears displayed significant kernel abortion (Fig. 2c,d), which is a key predictor of HI, suggesting that mutation in ZmPLD3 might trigger HI. These homozygous mutants were then used as males to pollinate the ZD958 tester line and haploid kernels were identified via seven polymorphic molecular markers randomly distributed across six chromosomes (Fig. 2e and Extended Data Fig. 7), flow cytometry (Fig. 2f and Extended Data Fig. 8) and phenotypic evaluations (Fig. 2g–i), respectively. The HIR of *zmpld3-1* and *zmpld3-2* were 0.96% and 0.85% respectively, which did not significantly differ from that of *mtl/zmpla1/nld*, whereas no haploids were detected among 2,041 individuals from hybrid offspring of ZD958 crossed with the WT (Fig. 3b and Supplementary Table 5). The diversity of the HIR of *mtl/zmpla1/nld* might be caused by the distinct genetic background and mutation types and a comparison of the HI ability of different genes should exclude these factors. In addition, there was no significant difference in HIR between *zmpld3-1* and *zmpld3-2*, which indicated that the absence of the second HKD domain of *ZmPLD3* was sufficient to trigger HI.

### *zmpld3* exhibited synergistic effects with *mtl/zmpla1/nld* on enhancing the HIR

**Extended Data Fig. 9:**
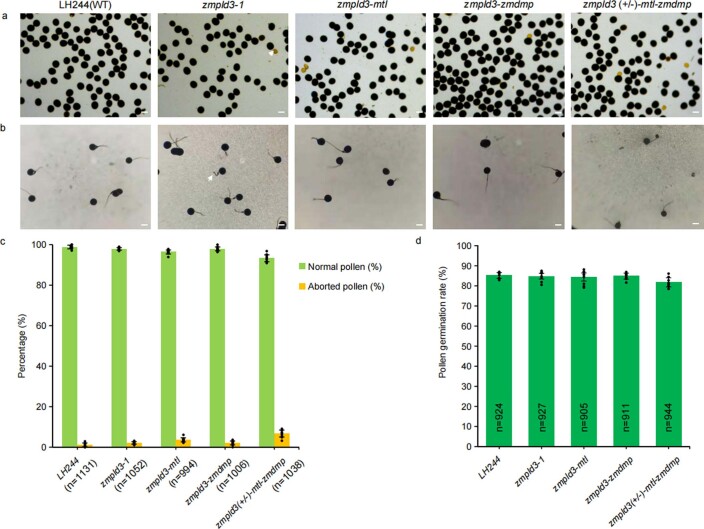
Pollen characteristics of *ZmPLD3*-related mutants compared to the wild type (WT). Detection results of pollen viability (a) and pollen germination (b) of LH244 (WT), *zmpld3-1*, *zmpld3-mtl*, *zmpld3-zmdmp* and *zmpld3 (+/-)-mtl-zmdmp*. The experiments were repeated ten times, and similar results were obtained. The arrowheads indicate aborted pollen. The arrows indicate germinated pollen. Scale bars, 15 μm. Statistical analysis of the percentage of two pollen viability types (c) and the pollen germination rate (d) for LH244 (WT), *zmpld3-1*, *zmpld3-mtl*, *zmpld3-zmdmp* and *zmpld3 (+/-)-mtl-zmdmp*. The values are the means ± s.d.; n, number of pollen grains.

To determine the effects between *zmpld3* and reported genes on HI, we generated double mutants of *zmpld3-mtl*, *zmpld3-zmdmp* and *mtl-zmdmp* via hybridization of the corresponding single-gene mutants. Genotyping of individuals in the segregating population from selfing of heterozygous double mutants revealed that there were different levels of segregation distortion among the three mutants. Mutations of *ZmPLD3* and *MTL/ZmPLA1/NLD* showed severe segregation distortion with similar degree, while mutation of *ZmDMP* showed slight segregation distortion in the segregating population (Supplementary Table 4). Afterward, F_1_ individuals derived from ZD958 ears pollinated by homozygous *zmpld3-mtl*, *zmpld3-zmdmp* or *mtl-zmdmp* were screened for their phenotypes related to HI. The statistical data revealed that *zmpld3* and *mtl/zmpla1/nld* exhibited synergistic effects, as the double mutants of *zmpld3* and *mtl/zmpla1/nld* could increase the HIR up to ~4% (Fig. 3b), whereas the HIR of the single mutation in *mtl/zmpla1/nld* was 1.2%. Meanwhile, the statistical data on average HIR indicated that there was no significant difference between the two genotypes of *mtl-zmdmp* and *zmpld3-mtl* (Fig. 3b). However, the average HIR of *zmpld3-zmdmp* did not significantly increase compared to that of *zmpld3*, while the HIR of *zmpld3* was significantly higher than that of *zmdmp* (Fig. 3b), implying that little interaction occurred between ZmPLD3 and ZmDMP. The aborted-kernel phenotype of the hybrid ears among the single mutants and double mutants showed concordant effects. The hybrid ears of *zmpld3-mtl* had significantly more aborted kernels than those of *zmpld3-1*, whereas no differences were observed between *zmpld3-zmdmp* and *zmpld3-1* (Fig. 3a,c). These findings indicated that *zmpld3* and *mtl/zmpla1/nld* had synergistic effects on improving the HIR rather than functional redundancy, although both ZmPLD3 and MTL/ZmPLA1/NLD belong to the phospholipase family. Notably, double mutant of *zmpld3-mtl* also resulted in a dramatic decrease in seed setting rate—down to ~70% (Fig. 3d). Pollen fertility via KI staining and pollen viability via pollen germination were measured for both the single and double mutants and no significant differences were observed between these mutants and the WT (Extended Data Fig. 9).

As both of *zmpld3* and *zmdmp* could enhance HIR in the presence of *mtl/zmpla1/nld*, it would be probable that combination of *zmpld3* and *mtl/zmpla1/nld* and *zmdmp* could generate an HI line with higher HIR. Among 2,725 individual plants from selfed progeny of heterozygous *zmpld3-mtl-zmdmp*, we did not obtain the homozygous triple mutant of *zmpld3-mtl-zmdmp*. Then we found that extreme segregation distortion was only observed for *zmpld3* mutation in the population (21 homozygous individuals in the 2,725 selfed offsprings), whereas the segregation-distortion pattern of *mtl/zmpla1/nld* mutation and *zmdmp* mutation in triple mutant background resembled that of corresponding single and double mutants (Supplementary Table 4). However, we identified three *zmpld3* (+/–)*-mtl-zmdmp* (mutation of *zmpld3* was heterozygous, while mutations of *mtl* and *zmdmp* were homozygous), of which pollens were used to pollinate ZD958 ears to evaluate their HIR; the results indicated that the HIR of *zmpld3* (+/–)*-mtl-zmdmp* was significantlly enhanced compared with that of *zmpld3-mtl* and *mtl-zmdmp* (Fig. 3b). Further screening of the inbred progeny of the three *zmpld3* (+/–)-*mtl*-*zmdmp* still did not yield homozygous triple mutant (Supplementary Table 6). It might be caused by pollen defects which were not detected by the standard pollen assays (Extended Data Fig. 9) or fertilization failure of gametes containing *zmpld3* mutation in triple mutant background. Therefore, getting the homozygous triple mutant could be very difficult or even impossible.

### ZmPLD3 localized to endoplasmic reticulum, plastids, Golgi apparatus and cytosol in maize protoplasts

**Fig. 4:**
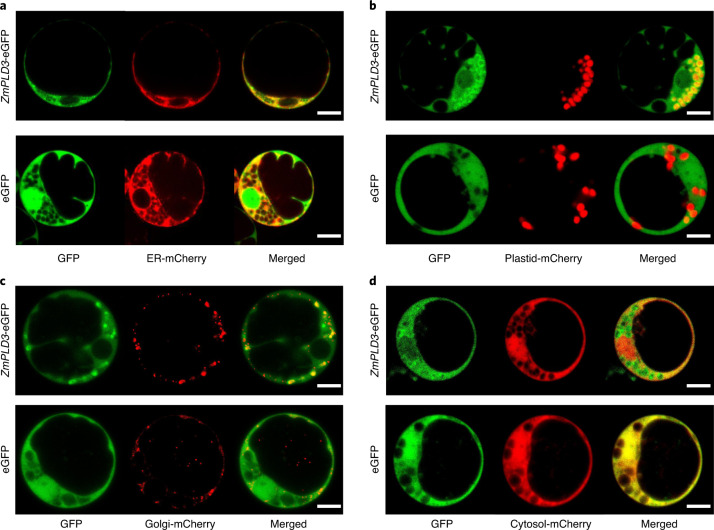
The subcellular localization of ZmPLD3. **a–d**, Transient co-expression of 35S::ZmPLD3-eGFP (at the top) or 35S::eGFP (at the bottom) with mCherry-labelled markers of the endoplasmic reticulum (ER) (**a**), plastids (**b**), Golgi apparatus (**c**) or cytosol (**d**) in maize protoplast cells, as determined by confocal laser-scanning microscopy. The experiments were repeated three times and similar results were obtained. Scale bars, 1 μm.

**Extended Data Fig. 10:**
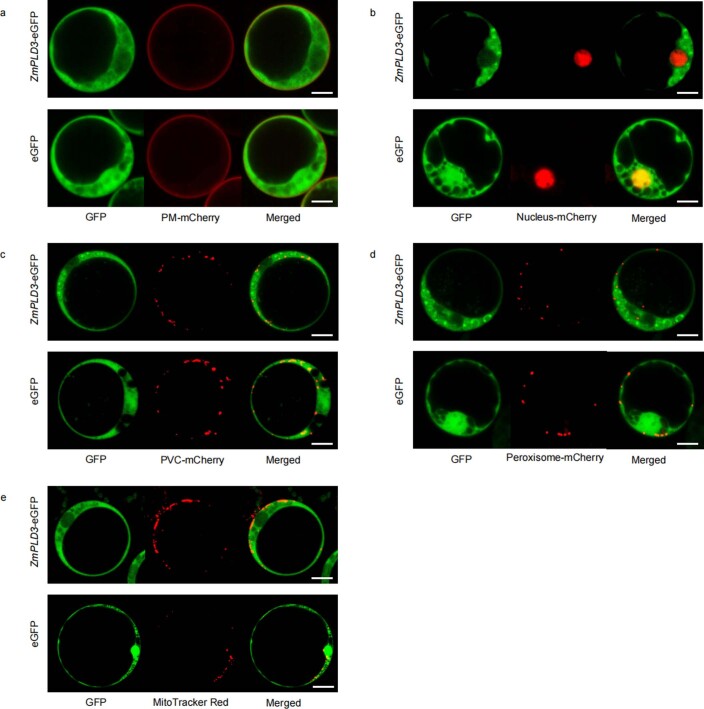
Co-expression of ZmPLD3 with different cellular compartment markers in maize protoplasts. a-e, Transient co-expression of 35S::ZmPLD3-eGFP (at the top) or 35S::eGFP (at the bottom) with mCherry-labelled markers of the plasma membrane (PM) (a), nuclear (b), prevacuolar compartment (PVC) (c), peroxisome (d) or mitochondrial red fluorescent probe MitoTracker Red (e) in maize protoplast cells, as determined by confocal laser-scanning microscopy. The experiments were repeated three times, and similar results were obtained. Scale bars, 1 μm.

It has been shown that α subfamily of PLD is present in soluble and membrane-associated fractions and its relative distribution between the two fractions depends on the tissues and developmental stages^29^. We used markers of different cellular compartments to characterize the subcellular localization of ZmPLD3 using maize protoplast system. The results indicated that ZmPLD3 could localize to the endoplasmic reticulum, plastids, Golgi apparatus and cytosol but probably not the plasma membrane, mitochondria, prevacuolar compartment, nuclear or peroxisome (Fig. 4 and Extended Data Fig. 10). Since transient protoplast expression could not identify the exact localization of ZmPLD3 in pollen, it would be worthwhile to conduct further studies such as stable transformation with its endogenous promoter to confirm the endogenous localization of ZmPLD3 in pollen.

### Transcriptome analysis of mutants involved in HI

**Fig. 5:**
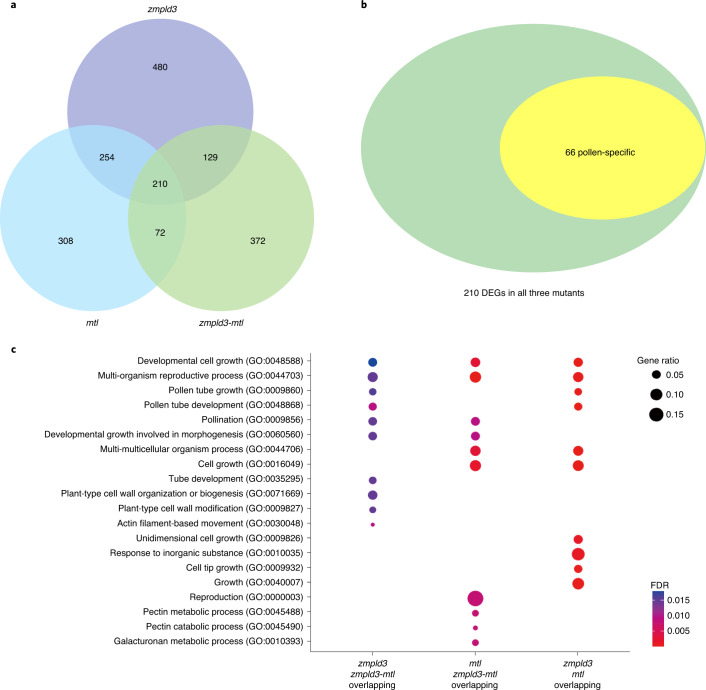
Transcriptional profiling of multiple pathways involved in haploid induction regulated by *zmpld3* and *mtl/zmpla1/nld*. **a**, Venn diagram illustrates the overlap of DEGs shared among *zmpld3*, *mtl/zmpla1/nld* and *zmpld3-mtl*. The data are derived from RNA-seq of *zmpld3* and *mtl/zmpla1/nld* and *zmpld3-mtl* pollen samples, each comprising two biologically independent replications. **b**, Venn diagram illustrates 66 pollen-specific DEGs shared among *zmpld3*, *mtl/zmpla1/nld* and *zmpld3-mtl*. **c**, GO analyses using a hypergeometric distribution of the top ten significantly enriched GO terms (FDR < 0.05) among the overlapping DEG sets was performed; those shared between *zmpld3* and *zmpld3-mtl*, between *mtl* and *zmpld3-mtl* and between *zmpld3* and *mtl* are shown﻿. Colour bar, FDR.

To identify genes involved in HI affected by the single and double mutations mentioned above, we conducted RNA-seq analysis on mature pollen collected from LH244 (WT), *zmpld3*, *mtl/zmpla1/nld* and *zmpld3-mtl*. Differentially expressed genes (DEGs) in these mutants were identified (false discovery rate (FDR) < 0.05; Fig. 5a and Supplementary Table 7). Gene ontology (GO) functional enrichment analysis of three types of overlapping DEGs among *zmpld3* and *mtl/zmpla1/nld* and *zmpld3-mtl* revealed that terms related to gametogenesis processes were enriched, such as pollen tube development and multi-organism reproductive processes (FDR < 0.05; Fig. 5c and Supplementary Tables 8–10). As the detection of pollen viability and germination rate showed no significant difference between the mutants and WT (Extended Data Fig. 9), the pollen of *zmpld3* might have altered the polar growth of pollen tubes or disrupted communication with female gametocytes; as such, investigating the changes in *zmpld3* pollen is worth further study. Furthermore, we investigated the pollen-specific DEGs in these mutants and 66 out of 210 overlapping DEGs in all three mutants were expressed specifically in pollen (Fig. 5b and Supplementary Table 11). Interestingly, we found that two pollen-specific DEGs (Zm00001d039429 and Zm00001d015414), which colocalized with previously reported QTLs for HI, *qhir2* and *qhir6*, respectively (Table 1), were predicted to be involved in the maintenance of pollen tube integrity^30^ or pollen tip growth during fertilization^31^. In addition, five pollen-specific DEGs acted in the maintenance of the degree of pectin methylesterification, the process of which is relevant to pollen tube growth or pollen tube attraction during fertilization^32,33^. These results suggested that genes involved in cell communication between the two gametophytes during double fertilization might be involved in HI. In addition, we have performed comparative analysis between the overlapping DEGs in our research and that of previous research of MTL^9^, which showed that only two genes of Zm00001d017246 and Zm00001d044227 from our overlapping DEGs in three mutants (*zmpld3*, *mtl/zmpla1/nld* and *zmpld3-mtl*) were identical to the previous research of MTL^1^. Zm00001d017246 was pollen-specific and annotated as lung seven transmembrane receptor family protein, whereas Zm00001d044227 was constitutively expressed and unannotated.

**Table 1:** Overlapping DEGs identified in *zmpld3*, *mtl* and *zmpld3-mtl* that colocalized with HI-related QTLs

Gene ID	Annotation	log_2_(fold change)			Note
		*zmpld3* versus WT	*mtl* versus WT	*zmpld3-mtl* versus WT	
Zm00001d039429^a^	RALF-like protein	1.93	1.98	2.09	bin3.02 (*qhir2*)
Zm00001d042634	Catalytic LigB subunit of aromatic ring-opening dioxygenase family	0.66	0.29	0.71	bin3.06 (*qhir3*)
Zm00001d042853	Unannotated	0.84	0.41	1.28	bin3.06 (*qhir3*)
Zm00001d042921	HSP20-like chaperones superfamily protein	−1.21	−1.13	−1.20	bin3.06 (*qhir3*)
Zm00001d015821^a^	Pectin lyase-like superfamily protein	0.27	0.58	0.75	bin5.04 (*qhir6*)
Zm00001d016510^a^	Cell wall/vacuolar inhibitor of fructosidase	0.97	1.28	1.54	bin5.04 (*qhir6*)
Zm00001d015414^a^	Leucine-rich repeat protein kinase family protein	0.31	0.45	0.53	bin5.04 (*qhir6*)
Zm00001d015284	Unannotated	0.43	0.43	0.64	bin5.04 (*qhir6*)
Zm00001d015638	Unannotated	0.82	1.31	0.92	bin5.04 (*qhir6*)
Zm00001d015272	Fucosyltransferase 1	0.39	0.49	1.13	bin5.04 (*qhir6*)
Zm00001d016066	Galactose oxidase/kelch repeat superfamily protein	−0.33	−0.31	−0.69	bin5.04 (*qhir6*)
Zm00001d016175	Myb-like HTH transcriptional regulator family protein	−0.86	−0.74	−1.62	bin5.04 (*qhir6*)
Zm00001d015628	Ribosomal protein S21 family protein	0.70	0.70	0.92	bin5.04 (*qhir6*)
Zm00001d018908	Unannotated	0.98	1.06	1.29	bin7.01 (*qhir7*)

^a^Pollen-specific genes.

## Discussion

We isolated ZmPLD3 by analysing publicly available RNA-seq data from multiple tissues, including pollen tissue and verified that the loss of function of ZmPLD3 triggered maternal HI in maize. Meanwhile, we have not found that this locus overlaps with previously reported QTLs underlying HI^3^, suggesting that the *ZmPLD3* locus might have not been selected by haploid-inducer breeders. Further research revealed that *zmpld3* and *mtl/zmpla1/nld* showed synergistic effects rather than functional redundancy on improving HI, which implied that ZmPLD3*-*mediated pathways might interact synergistically with those of MTL/ZmPLA1/NLD in HI. Intracellular localization of ZmPLD3 indicated the possibility that endomembrane transport signalling and lipid metabolism might also be involved in HI. It has been speculated that the mutation in *ZmDMP* impaired double fertilization and created additional single-fertilization events, thereby enhancing HIR in the presence of *mtl/zmpla1/nld*^6,34^. Considering the distinct effects of *ZmPLD3* and *ZmDMP* on HI (Fig. 3), the mechanism underlying HI is more complex than the oversimplified accumulation of components of distinct regulatory pathways. Overall, we inferred that ZmPLD3 might function in distinct pathways paralleling with those of MTL/ZmPLA1/NLD, giving rise to their synergistic effects on HI.

Previous studies have provided compelling evidence for the hypothesis concerning genome elimination in HI^35–38^; mutations in *MTL/ZmPLA1/NLD* might cause all or partial genome instability and continuous chromosome fragmentation. Successful screening of haploid progeny via CRISPR-Cas9-induced mutations through HI editing (HI-Edit)^37^ or haploid-inducer mediated genome editing (IMGE)^38^ directly proved that a transient fusion state of sperm and egg cell genomes happened before paternal genome elimination. Although HI-Edit/IMGE enabled the universal application of genome-editing technologies in commercial crop improvement, the average editing efficiency of haploids by the HI-Edit/IMGE system was ~3–4% in maize. In combination with the low HIR of the haploid inducers, the HI-Edit/IMGE system is still inefficient for practical breeding processes. Thus, increasing chromosome elimination-mediated HI would theoretically improve the efficiency of HI-Edit/IMGE. Our RNA-seq data revealed that multiple genes involved in pathways of cell communication between male gametophytes and female gametocytes were significantly changed in *zmpld3* and *mtl/zmpla1/nld* and the altered expression of these genes might lead to male-specific developmental defects and genome elimination during double fertilization, implying that uniparental chromosome elimination might be enhanced in the *zmpld3* and *mtl/zmpla1/nld* double mutants. More data are needed to verify whether altered composition of the pollen of phospholipase-related mutants triggers chromosome fragmentation in sperm cells during fertilization.

In our present study, a reverse genetic strategy such as that for ZmPLD3 represented a novel approach to expand genetic resources for the potential of breeding super haploid inducers. Further studies on pollen-specific DEGs involved in HI will not only contribute to elucidating the molecular network of HI but also offer a high probability that pyramiding these genes via genome editing would generate new haploid inducers with higher HIR. However, additional study is needed to determine whether and how the enhancement of HIR would lead to high cost of reproductive fitness. Moreover, high conservation of ZmPLD3 in cereals might extend its applications to other crops.

## Methods

### Identification of phospholipases in maize and phylogenetic analysis

The amino acid sequences of the members of the phospholipase family in *A. thaliana* and rice were used as queries to search for homologous sequences in MaizeGDB (http://www.maizegdb.org). Putative maize phospholipases were further confirmed for the presence of the conserved domains associated with different phospholipase classes (PLA, PLC and PLD) by scanning sequences through the SMART (http://smart.embl-heidelberg.de/), Pfam (http://pfam.sanger.ac.uk/search) and InterPro (http://www.ebi.ac.uk/interpro/) online databases. All maize phospholipases are listed in Supplementary Table 1. The PLD subfamily in *A. thaliana*, rice and maize was used to construct a phylogenetic tree with the neighbour-joining method by using MEGA-X software. The PLD proteins used to construct the phylogenetic tree are shown in Supplementary Table 3.

### Expression and quantitative real-time PCR analysis of *ZmPLD3*

We used public RNA-seq data from different tissues^27^ and downloaded them from the National Center for Biotechnology Information (NCBI) database (http://www.ncbi.nlm.nih.gov/) to identify the expression characteristics of *ZmPLD3*. Total RNA from 1-mm-long immature anthers, 2-mm-long immature anthers, 3-mm-long immature anthers, 4-mm-long immature anthers and mature pollen from LH244 was extracted using TRIzol reagent (15596026, Invitrogen) and then reverse transcribed into complementary DNA. *ZmPLD3*-specific primers were designed using Primer-BLAST (the sequences of which are listed in Supplementary Table 12). Quantitative real-time PCR using SYBR Green PCR mix (RR820Q, TaKaRa) was performed with an ABI 7500 system according to the manufacturers’ instructions. Transcript abundance was compared with that of an endogenous control (NADPH) to standardize the starting cDNA amounts and relative expression of *ZmPLD3* in each tissue compared with that of the control was calculated via the 2^−∆∆CT^ method.

### Subcellular localization of ZmPLD3

The full-length coding sequence of ZmPLD3 without the stop codon was cloned into pCAMBIA1300-35S::eGFP for subcellular localization analysis. The resulting pCAMBIA1300-35S::ZmPLD3-eGFP vector was subsequently cotransformed with AtHDEL-mCherry (an endoplasmic reticulum marker)^39^, AtSYP61-mCherry (a Golgi marker)^40^, WxTP-mCherry (a plastid marker)^41^, AtCBL1-mCherry (a plasma membrane marker)^42^, AtAHL22-mCherry (a nuclear marker)^43^, AtVSR2-mCherry (a prevacuolar compartment marker)^44^, AtPTS1-mCherry (a peroxisome marker)^45^ or free mCherry (a cytosol marker)^46,47^ into maize protoplasts. A pCAMBIA1300-35S::eGFP unmodified vector was also cotransformed with the same markers into maize protoplasts, which served as controls. After culturing at 28 °C for 18 h, fluorescent signals were detected using a confocal microscope (Zeiss 880). For observation of mitochondrial localization, only pCAMBIA1300-35S::ZmPLD3-eGFP vector or pCAMBIA1300-35S::eGFP unmodified vector was transformed into maize protoplasts and after culturing at 28 °C for 18 h, protoplasts were incubated with the diluted mitochondrial red fluorescent probe MitoTracker Red (25 nM) (40741ES50; YEASEN) for ~30 min and then detected with confocal microscope.

### Plant materials and growth conditions

The transformable line LH244 was provided by the US Department of Agriculture (https://npgsweb.ars-grin.gov/gringlobal/search.aspx). All the maize materials were grown in a greenhouse under a 16 h/8 h light/dark photoperiod at 28 °C/24 °C, with the relative humidity held constant. Transgenic plants and ZD958 tester plants were grown in open field in the summer and all the test crosses (include all the mutants and wild type) were carried out in open field in Shangzhuang Experimental Base of China Agricultural University in the same season in Beijing, China. Besides, all the pollinated ears were included in the analysis of kernel abortion and HIR.

### Gene editing and detection analysis of edited mutations

The CRISPR-Cas9 system was used to generate *zmpld3*, *mtl* and *zmdmp* mutants. The sequences of the gRNAs of each single-gene mutant were inserted into a binary vector (pCAMBIA3301) expressing Cas9 and gRNA^48^. The primers used for vector construction are listed in Supplementary Table 12. Embryos of LH244 (at 12 d after pollination (DAP)) were used for *Agrobacterium*-mediated transformation experiments. In brief, the vectors were transformed into strain EHA105 and a single clone was cultured in liquid YEP media. The prepared *Agrobacterium* containing the target vectors was used to infect embryos at 12 DAP (1.5–1.8 mm) for 30 min, followed by culturing at 22 °C for 3 d. The cultured embryos were then transferred to selection media and allowed to grow at 28 °C for 2 weeks in darkness. All the calli on the selection media were moved to regeneration media for shooting and rooting. Positive transgenic events were identified via herbicide resistance and verified by DNA sequencing at the seedling stage. Knockout lines with frameshift mutations were transferred to a greenhouse and backcrossed twice to LH244 and then self-pollinated to acquire homozygous knockout mutants without transgenic elements of CRISPR–Cas9 through bialaphos resistance gene (bar) strip tests and PCR sequencing analysis.

### Characterization of HI-related phenotypes

HI was performed by crossing different knockout lines with the tester line ZD958. WT LH244 was used to pollinate ZD958 as a control. The seedlings of the F_1_ population were screened for ploidy via PCR assays. The haploid candidates were determined on the basis of polymorphic markers with polymorphisms between the receptor line and ZD958; the candidates which had only an amplified product corresponding to the maternal line were screened out. These haploid candidates were then further confirmed via flow cytometry (ploidy analysis) and the candidates with peaks similar to those of standard haploids were considered as true haploids^1^. Moreover, the candidates were grown to observe their phenotypes. Compared with the diploid controls, all these haploids were shorter in height, had narrower leaves (the 12th leaf of each plant was measured) and had smaller anthers. In addition to the HIR, the rate of endosperm aborted kernels (EnAR) was also measured according to the methods of Liu et al.^10^. Statistical analysis via the Mann–Whitney test was used to assess significance by SigmaPlot 12.5 software and the resulting *P* values are noted in the figures and captions.

### Determination of pollen viability and germination

Fresh pollen samples of LH244, *zmpld3-1*, *zmpld3-2*, *zmpld3-mtl* and *zmpld3-zmdmp* were collected between 10:00 and 11:00 in the greenhouse and three biological replicates were included for each collection. Pollen viability was measured via 1% KI/I_2_ solution; after 5 min, the viability was checked by microscope examination. Pollen germination assays were conducted on pollen germination media (10% sucrose, 0.01% boric acid, 0.1% yeast extract, 10 mM CaCl_2_, 50 μM KH_2_PO_4_, 15% polyethylene glycol 4000)^49^; after 1 h of incubation at 28 °C, the pollen germination rate was evaluated via microscopy to determine the percentage of pollen with elongated tubes.

### RNA-seq profiling and analysis

Total RNA of the mature pollen was extracted using TRIzol reagent (Invitrogen) and two biological replicates for each sample were collected for RNA extraction. The mRNA-seq libraries were constructed with an mRNA-seq library preparation kit (Vazyme) and sequenced on an Illumina NovaSeq platform for 150-nucleotide paired-end reads. The B73 reference genome (RefGen_v4)^50^ sequence was downloaded from http://ensembl.gramene.org/Zea_mays/Info/Index. After removing low-quality reads using FASTP (v.0.20.1; http://github.com/OpenGene/fastp), the raw reads were aligned to the B73 reference genome using HISAT2 (v.2.2.1, http://daehwankimlab.github.io/hisat2/). The uniquely mapped reads were used to obtain read counts of each gene in the B73 reference genome by parsing the alignment output files from HISAT2 and then normalizing the resulting read counts via fragments per kilobase of exon model per million mapped fragments (FPKM) using Cufflinks (v.2.2.1)^51^ to measure the gene expression levels. The agriGO^52^ online tool was subsequently used to perform a GO analysis. For identification of pollen-specific genes, we used publicly available RNA-seq data^27^ of 23 different tissue samples downloaded from the NCBI database (http://www.ncbi.nlm.nih.gov/). The expression levels across all of the samples were normalized according to the log_2_(FPKM + 0.01). Using normalized expression levels, we then calculated *z*-scores of the given genes in pollen and compared them with those of genes in other tissue samples. A gene was determined to be expressed specifically in pollen if it had a *z*-score >3, its FPKM ≥ 1 and its highest FPKM value was for a gene expressed in pollen. The R package DESeq2 (http://www.bioconductor.org/packages/release/bioc/html/DESeq2.html) was used to identify DEGs in each mutant compared with the WT. *P* values of all the statistical tests were adjusted to *q* values and an FDR of 5% was applied. The RNA-seq data have been submitted to the NCBI database and the accession number is PRJNA723300.

### Reporting Summary

Further information on research design is available in the Nature Research Reporting Summary linked to this article.

## Supplementary information

Reporting Summary

Supplementary Tables Supplementary Table 1. Summary of phospholipase genes in maize. Supplementary Table 2. Transcript levels (fragments per kilobase of exon model per million mapped fragments (FPKM)) of phospholipases in maize. Supplementary Table 3. Summary of phospholipase D (PLD) genes in *Zea mays*, *Oryza sativa* and *Arabidopsis thaliana*. Supplementary Table 4. Segregation of *zmpld3*, *mtl/zmpla1/nld*, and *zmdmp* in the selfed progeny of different heterozygous mutants. Supplementary Table 5. Phenotypic data related to haploid induction (HI) of plants with homozygous mutations in *ZmPLD3*, *MTL/ZmPLA1/NLD* and *ZmDMP*. Supplementary Table 6. Segregation of *zmpld3* in the inbred progeny of *zmpld3 (+/-)-mtl-zmdmp*. Supplementary Table 7. Detailed information on differentially expressed genes (DEGs) from mutants (*zmpld3*, *mtl/zmpla1/nld*, *zmpld3-mtl*) based on RNA sequencing (RNA-seq) (fragments per kilobase of exon model per million mapped fragments (FPKM)). Supplementary Table 8. Gene Ontology (GO) term enrichment analysis of the differentially expressed genes (DEGs) between *zmpld3* and *zmpld3-mtl*. Supplementary Table 9. Gene Ontology (GO) term enrichment analysis of the differentially expressed genes (DEGs) between *mtl/zmpla1/nld* and *zmpld3-mtl*. Supplementary Table 10. Gene Ontology (GO) term enrichment analysis of the differentially expressed genes (DEGs) between *zmpld3* and *mtl/zmpla1/nld*. Supplementary Table 11. Sixty-six pollen-specific differentially expressed genes (DEGs) overlapping among *zmpld3*, *mtl/zmpla1/nld* and *zmpld3-mtl*. Supplementary Table 12. Primers used in this study.

## Data Availability

The RNA-seq data of this study have been deposited in the NCBI SRA BioProject database under accession number PRJNA723300. Source data are provided with this paper. All other data are available from the corresponding author on reasonable request.

## Source data

Source Data Fig. 2 Unprocessed gels of Fig. 2e.
Source Data Extended Data Fig. 7 Unprocessed gels.
