# Supplementary material for: Loss-of-function alleles of ZmPLD3 cause haploid induction in maize
Source: Nat Plants. 2021 Dec 9;7(12):1579–88. doi: 10.1038/s41477-021-01037-2 (PMC8677622; doi:10.1038/s41477-021-01037-2)
Supplement: Supplementary file 1 — Reporting Summary [file 41477_2021_1037_MOESM1_ESM.pdf]

Corresponding author(s): Weibin Song

Last updated by author(s): Oct 25, 2021

Reporting Summary

Nature Research wishes to improve the reproducibility of the work that we publish. This form provides structure for consistency and transparency in reporting. For further information on Nature Research policies, see our Editorial Policies and the Editorial Policy Checklist.

Statistics

For all statistical analyses, confirm that the following items are present in the figure legend, table legend, main text, or Methods section.

n/a	Confirmed	
☐	☒	The exact sample size (*n*) for each experimental group/condition, given as a discrete number and unit of measurement
☐	☒	A statement on whether measurements were taken from distinct samples or whether the same sample was measured repeatedly
☐	☒	The statistical test(s) used AND whether they are one- or two-sided<br>*Only common tests should be described solely by name; describe more complex techniques in the Methods section.*
☒	☐	A description of all covariates tested
☒	☐	A description of any assumptions or corrections, such as tests of normality and adjustment for multiple comparisons
☐	☒	A full description of the statistical parameters including central tendency (e.g. means) or other basic estimates (e.g. regression coefficient) AND variation (e.g. standard deviation) or associated estimates of uncertainty (e.g. confidence intervals)
☐	☒	For null hypothesis testing, the test statistic (e.g. *F*, *t*, *r*) with confidence intervals, effect sizes, degrees of freedom and *P* value noted<br>*Give P values as exact values whenever suitable.*
☒	☐	For Bayesian analysis, information on the choice of priors and Markov chain Monte Carlo settings
☒	☐	For hierarchical and complex designs, identification of the appropriate level for tests and full reporting of outcomes
☒	☐	Estimates of effect sizes (e.g. Cohen's *d*, Pearson's *r*), indicating how they were calculated

Our web collection on statistics for biologists contains articles on many of the points above.

Software and code

Policy information about availability of computer code

Data collection	Becton Dickinson FACSCalibur system
Data analysis	Phylogenetic analysis: MEGA-X version 10.2.2,<br>Ploidy analysis: Becton Dickinson CellQuest Pro<br>Confocol microscopy: Zeiss 880<br>RNA-seq analysis: FASTP version 0.20.1, HISAT2 version 2.2.1, Cufflinks version 2.2.1, DESeq2<br>GO analysis: agriGO version 2.0<br>Other: Excel 2019, SigmaPlot 12.5

For manuscripts utilizing custom algorithms or software that are central to the research but not yet described in published literature, software must be made available to editors and reviewers. We strongly encourage code deposition in a community repository (e.g. GitHub). See the Nature Research guidelines for submitting code & software for further information.

Data

Policy information about availability of data

All manuscripts must include a data availability statement. This statement should provide the following information, where applicable:

- Accession codes, unique identifiers, or web links for publicly available datasets
- A list of figures that have associated raw data
- A description of any restrictions on data availability

All sequencing data have been submitted to the NCBI Sequence Read Archive (SRA; http://www.ncbi.nlm.nih.gov/sra) under accession number PRJNA723300.

Field-specific reporting

Please select the one below that is the best fit for your research. If you are not sure, read the appropriate sections before making your selection.

☒ Life sciences ☐ Behavioural & social sciences ☐ Ecological, evolutionary & environmental sciences

For a reference copy of the document with all sections, see nature.com/documents/nr-reporting-summary-flat.pdf

Life sciences study design

All studies must disclose on these points even when the disclosure is negative.

Sample size	No statistical methods were used to predetermine sample size. Sample sizes were chosen based on availability of seeds, space considerations in the greenhouse and the desire to get statistically significant data to support meaningful conclusions.
Data exclusions	No data was excluded.
Replication	The number of Replications is indicated in the manuscript. Main conclusions were confirmed in different assays (qRT-PCR assay, HI-related phenotypic characterization assay, pollen viability and germination assay, Subcellular localization assay, RNA-seq assay). All the experiments were repeated at least three times independently with similar results.
Randomization	Experimental groups of different mutants and wild type were planted randomly in the field to mitigate potential variables, which include the availability of water, light and air flow.
Blinding	Data collection, such as the measurements of haploid induction rate were conducted by a third person who was blinded to sample identities.

Reporting for specific materials, systems and methods

We require information from authors about some types of materials, experimental systems and methods used in many studies. Here, indicate whether each material, system or method listed is relevant to your study. If you are not sure if a list item applies to your research, read the appropriate section before selecting a response.

Materials & experimental systems

n/a	Involved in the study	
☒	☐	Antibodies
☒	☐	Eukaryotic cell lines
☒	☐	Palaeontology and archaeology
☒	☐	Animals and other organisms
☒	☐	Human research participants
☒	☐	Clinical data
☒	☐	Dual use research of concern

Methods

n/a	Involved in the study	
☒	☐	ChIP-seq
☐	☒	Flow cytometry
☒	☐	MRI-based neuroimaging

Flow Cytometry

Plots

Confirm that:

☒ The axis labels state the marker and fluorochrome used (e.g. CD4-FITC).

☒ The axis scales are clearly visible. Include numbers along axes only for bottom left plot of group (a 'group' is an analysis of identical markers).

☐ All plots are contour plots with outliers or pseudocolor plots.

☐ A numerical value for number of cells or percentage (with statistics) is provided.

Methodology

Sample preparation	First, take a small amount of fresh leaf tissue (~1 gram), and mechanically chop it to release the nuclei into a nuclei isolation buffer (2ml). Next, remove large debris by filtration, and centrifuge (1000 rev/min) for 5 minutes to collect sediment. After that, use PI (propidium iodide) for fluorescent staining of nuclear DNA. Finally, put the samples into dark for20 min waiting for flow cytometric analysis.
Instrument	Becton Dickinson FACSCalibur system

Software	Becton Dickinson CellQuest Pro
Cell population abundance	After filtration, cells in the suspension were performed experimental procedures. Around 10000 cells were analyzed per sample.
Gating strategy	Bulk cell population was used in flow cytometry experiment. LH244, with the first signal peak at ~400 (FL2-A value), was used as a diploid control. Samples with the first signal peak at ~200 (FL2-A value) were deemed to be haploids.

☒ Tick this box to confirm that a figure exemplifying the gating strategy is provided in the Supplementary Information.
