# Supplementary figures and images for: Loss-of-function alleles of ZmPLD3 cause haploid induction in maize
Source: Nat Plants. 2021 Dec 9;7(12):1579–88. doi: 10.1038/s41477-021-01037-2 (PMC8677622; doi:10.1038/s41477-021-01037-2)

Fig.2e (original)

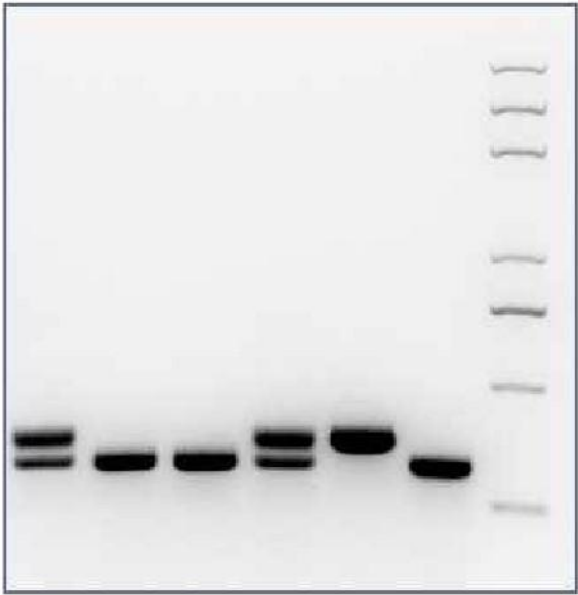

Supplement: Source Data Fig. 2 — Unprocessed gels of Fig. 2e. [file 41477_2021_1037_MOESM3_ESM.pdf]
