# Supplementary material for: Loss-of-function alleles of ZmPLD3 cause haploid induction in maize
Source: Nat Plants. 2021 Dec 9;7(12):1579–88. doi: 10.1038/s41477-021-01037-2 (PMC8677622; doi:10.1038/s41477-021-01037-2)

Extended Data Fig. 7-chr1-275.3(original)

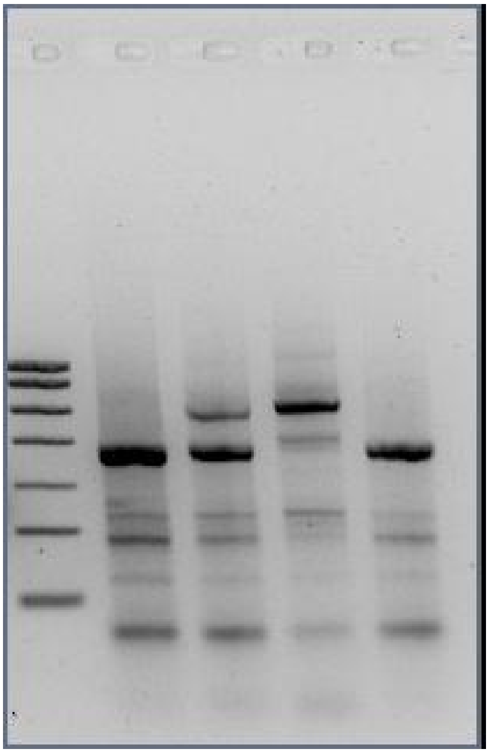

Extended Data Fig. 7-chr3-26.4(original)

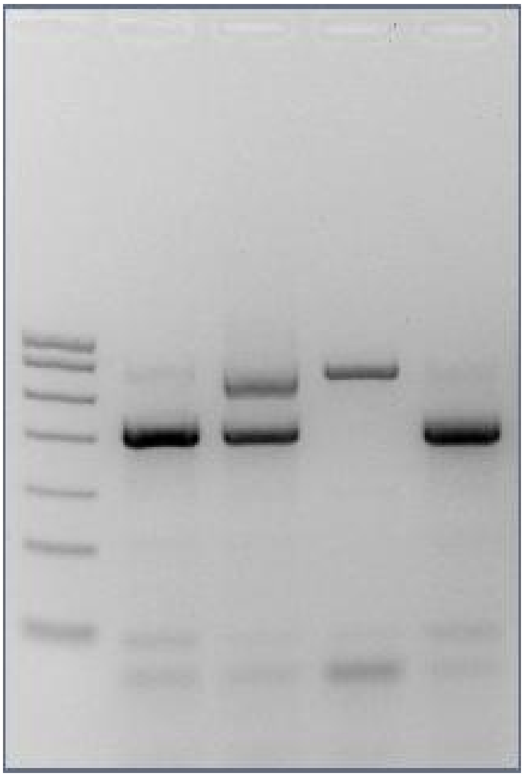

Extended Data Fig. 7-chr4-222.7(original)

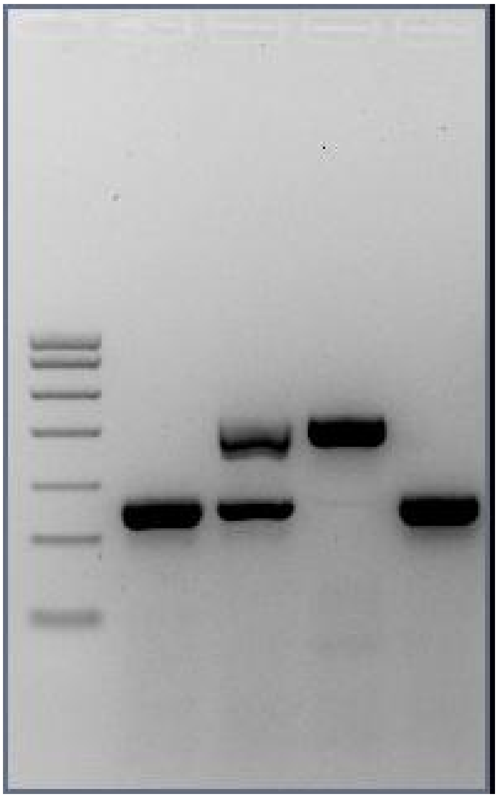

Extended Data Fig. 7-chr5-76.6(original)

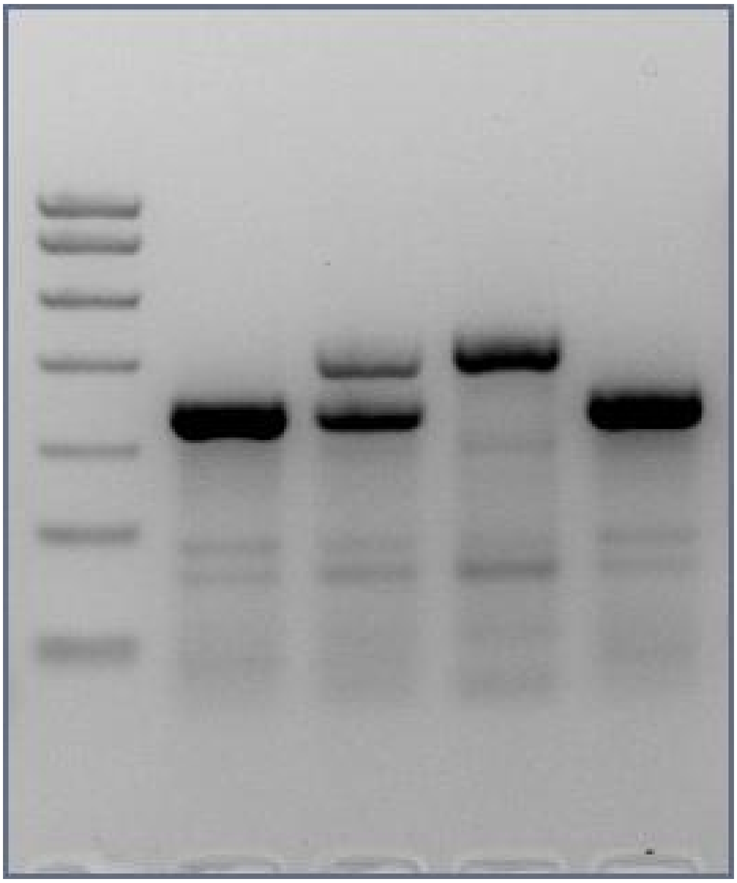

Extended Data Fig. 7-chr5-123.5(original)

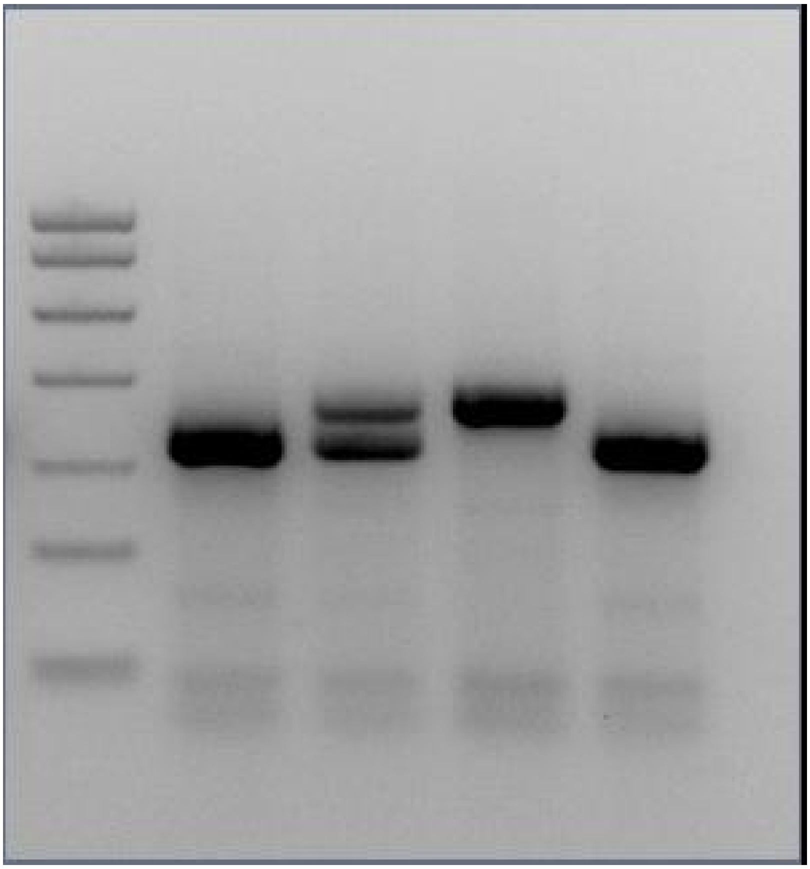

Extended Data Fig. 7-chr7-20.7(original)

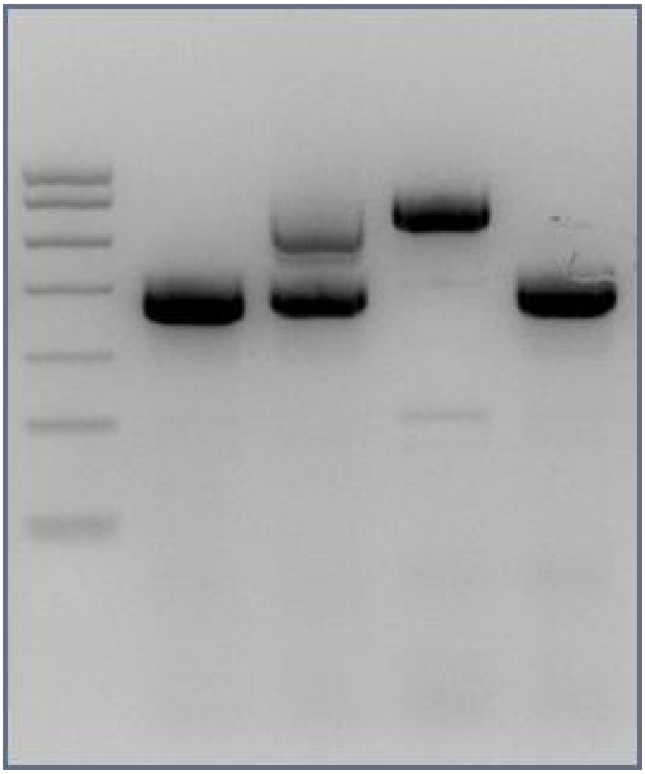

Extended Data Fig. 7-chr9-81(original)

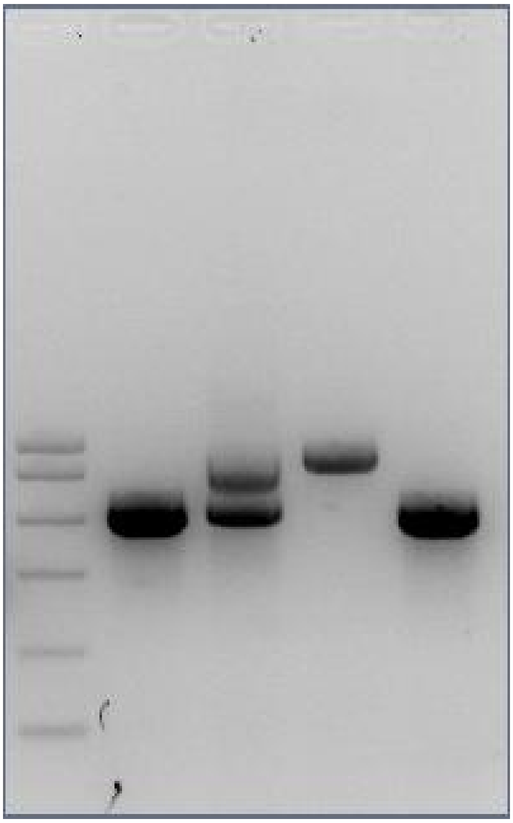

Supplement: Source Data Extended Data Fig. 7 — Unprocessed gels. [file 41477_2021_1037_MOESM4_ESM.pdf]
